# Validation of computational fluid dynamics models for airway deposition with SPECT data of the same population

**DOI:** 10.1038/s41598-024-56033-1

**Published:** 2024-03-06

**Authors:** Hosein Sadafi, Navid Monshi Tousi, Wilfried De Backer, Jan De Backer

**Affiliations:** 1 Fluidda N.V., Groeningenlei 132, 2550 Kontich, Belgium; 2 Department of Respiratory Medicine, University of Antwerp, Antwerpen, Belgium; 3 Fluidda Inc., 228 East 45th Street, 9th Floor, Suite 9E, New York, NY 10017 USA

**Keywords:** Respiratory tract diseases, Computational science, Mechanical engineering

## Abstract

This study compared computational fluid dynamic (CFD) model predictions on aerosol deposition in six asthmatic patients to the in-vivo results of the same group. Patient-specific ventilation and internal air distribution were prescribed using inspiratory and expiratory CT scans of each patient, accounting for individual lobar air flow distribution. Moreover, the significant influence of realistic mouth and throat geometries on regional deposition is demonstrated. The in-vivo data were obtained from single photon emission computed tomography (SPECT) in 6 subjects with mild asthma selected from a database of historical clinical trials. The governing flow and particle tracking equations were solved numerically using a commercial CFD tool, and the modeled deposition results were compared to the SPECT data. Good agreement was found between the CFD model, applying k-ω SST turbulence model, and SPECT in terms of aerosol deposition. The average difference for the lobar deposition obtained from CFD model and SPECT/CT data was 2.1%. The high level of agreement is due to applying patient specific airway geometries and inspiratory/expiratory CT images, anatomical upper airways, and realistic airway trees. The results of this study show that CFD is a powerful tool to simulate patient-specific deposition if correct boundary conditions are applied and can generate similar information obtained with functional imaging tools, such as SPECT.

## Introduction

More than one billion people worldwide suffer from asthma, cystic fibrosis (CF) and other chronic respiratory diseases^1^. The medications prescribed for these diseases are often inhaled by patients and may be administered by various inhalers. The effectiveness of inhaled medications often depends on where the inhaled drug deposits in a patient's airway. For example, if the medication is deposited in the oropharynx, it will not be effective in improving lung function and may cause oral infections.

Single photon emission computed tomography (SPECT) and gamma scintigraphy are the techniques used in practice to visualize drug deposition in the respiratory system^2^. However, these methods involve the use of radio labelled tracers, which may require specialized equipment and trained personnel. Additionally, gamma scintigraphy can only produce 2D images, which may limit the understanding of particle positioning in the airways^3,4^.

Computational fluid dynamics (CFD) may provide an alternative solution to the limitations of SPECT and gamma scintigraphy. This method relies on computer modeling based on physical laws and does not require the use of tracers and offers a 3D solution. Several researchers investigated aerosol deposition in human airway using CFD. Ciciliani et al.^5^ compared the effectiveness of different inhalers and assessed the effect of laminar and turbulent flow regimes on regional deposition. They showed that turbulent flow resulted in a higher deposition in the first 14 generations of the lung. The effect of geometrical variation of airways has been the focus of many researches, amongst all, Poorbahrami et al.^6^ studied the effect of age on deposition by investigating the airways of three age groups, infants, children and adults. The conclusion was the dosimetry in young subjects (especially infants) cannot be estimated by scaling adults' airway volume, but rather must be measured according to their own specific airways. Wedel et al.^7^ studied three different realistic, subject-specific replicas of the human lung and realized that the respiratory geometrical variations alters the local deposition patterns significantly, especially in the laryngeal airway region. Williams et al.^8^ explored the impact of airway structure as well as the breathing profile and particle size effect. They noticed for 4 µm and 10 µm particle sizes, the size, location, and intensity of local deposition hotspots varied with patient inhalation and airway shape, indicating the importance of using patient-specific image-based models to predict therapeutic outcomes in respiratory studies. Furthermore, they found the influence of physical sub-models such as particle–particle interactions and particle–wall lubrications were minor on deposition ratios across each lobe. Atzeni et al.^9^ compared the transient and steady state simulations. Using former, they were able to properly capture flow physics occurring in upper airway region that found to be influential in the total deposition. They observed that a steady state model (constant airflow) underestimates total deposition.

The acceptance of CFD results for clinical applications is contingent upon adherence to a set of established criteria, including validation of the CFD model through comparison to experimental data or in-vivo measurements^10^. Previous studies have validated CFD models against in-vivo datasets. For example, De Backer et al.^11^ assessed the accuracy of a steady state CFD model against a SPECT/Computed Tomography (CT) approach for the study of drug deposition in the airways of asthma patients when administered via nebulizer. Their comparison revealed the importance of incorporating real patient airway geometries and appropriate boundary conditions. Applying patient specific conditions, they achieved an average difference for the internal airflow distribution of less than 3% for CFD and CT versus SPECT/CT. Similarly, Tian et al.^12^ compared their CFD results with a different set of in-vivo data. They considered a whole lung model by combining an idealized mouth-throat and upper tracheobronchial airways to a stochastic individual pathway. The error of their results was approximately 10% compared to in-vivo results. In a later study, Kannan et al.^13^ utilized the same experimental data and compared the results with their CFD simulations on two idealized airways. They spotted that the airway incorporating the trachea’s ring structure led to higher airflow vorticity and consequently higher deposition level than the ring-less one, highlighting the need for using a realistic airway in CFD modeling.

Despite advancements in CFD techniques, comparability against patient-specific airways and boundary conditions remains inadequate. The studies in literature typically compare CFD simulations of an idealized airway to in-vivo scintigraphy of a separate airway^12–14^. This limitation emphasizes the need for further research utilizing patient-specific models in CFD simulations, and comparing the results to corresponding experimental studies, in order to understand the capabilities and limitations of CFD in predicting drug delivery within the human airway. The objective of this study is to compare the results of a CFD model for pharmaceutical aerosol deposition throughout the lungs of asthmatic patients with their respective SPECT datasets. To the authors knowledge, this study is the first to compare a transient CFD model predictions of aerosol deposition in six asthmatic patients with real in-vivo data of the same patients. Using patient-specific boundary conditions derived from inspiratory and expiratory CT scans, the individual lobar airflow distribution was applied in the model. Additionally, it underscores the significant impact of realistic mouth and throat structures on how inhaled particles deposit within the airways.

## Materials and method

The SPECT data used for comparison with the CFD model were extracted from the patients previously enrolled in a single study described in Ref.^11^. The Institutional Review Board at University Hospital Antwerp approved the study protocol of the SPECT tests, and all methods and research were performed in accordance with relevant regulations. All patients undergoing the SPECT tests gave their signed informed consent. Six subjects with mild asthma (three men; three women; mean ± SD age 46 ± 17 years) were selected from a database of historical study participants based on the availability of thin-slice, volumetric, paired-inspiratory expiratory MDCT data, contemporaneous SPECT deposition data, and clinical severity information. The SPECT/CT tracer and CT images were re-registered and fused at corresponding locations. In that experiment, the utilized radio-labeled aerosol was technetium 99 m (${}^{99m}TC$) pentetic acid, commonly used across Europe in clinical practices. Various nebulizers designed for ${}^{99m}TC$ pentetic acid are available in the market, and for this study, the SmartVent nebulizer was utilized to administer radiolabeled aerosols to the patients. The aerosols had an average diameter of approximately 1.32 µm and a density of 1015 kg/m^3^. Administration occurred through a mouthpiece while the patient's nose was blocked, allowing for slow tidal breathing. For each patient, two scans were captured at Functional Residual Capacity (FRC) and Total Lung Capacity (TLC) levels which are used to apply patient-specific boundary conditions in CFD model. More information on the SPECT settings and the patient list is available in Ref.^11^.

To perform CFD simulations that account for both airflow and particle motion in patient-specific airways, the Eulerian–Lagrangian framework is employed. For the Eulerian (fluid) phase, the Reynolds-Averaged Navier–Stokes (RANS) is implemented since it has been shown to be a reliable choice in particle tracking studies^15,16^. Additionally it is computationally more affordable when compared to Large Eddy simulation (LES) or direct numerical simulation (DNS) models. The continuity and momentum equations described with RANS are as follows:

1 $$\nabla \cdot \overline{u}=0,$$

2 $$\rho \frac{\partial \overline{u}}{\partial t}+\rho \left(\overline{u}\cdot \nabla \right)\overline{u}=-\nabla \overline{p}+\nabla \cdot \left(\mu \nabla \overline{u}-\rho \overline{u\otimes u}\right),$$

where $t$ is time, and $\overline{u}$ and $\overline{p}$ represent the ensemble average of velocity and pressure, respectively; $\rho$ and $\mu$ are the fluid density and dynamic viscosity and $- \rho \overline{u\otimes u}$ is the Reynolds stress tensor that has to be modelled using a turbulence model. The k-ω with shear stress transport (SST) was adapted, which employs two transport equations for modelling the Reynolds stresses^17^.

Regarding the discrete phase (particles), the equations are written in a Lagrangian framework which consist of the force balance over a particle:

3 $$\frac{d{x}_{p}}{dt}={u}_{p},$$

4 $${m}_{p}\cdot \frac{d{u}_{p}}{dt}=\sum {F}_{i}={F}_{D},$$

where ${x}_{p}$ denotes the particle position, ${u}_{p}$ and ${m}_{p}$ are the particle velocity and mass, and the term $\sum {F}_{i}$ represents the sum of all relevant forces acting on particles. The effect of forces, such as gravitational, Brownian motion and buoyancy found to be negligible^18,19^. Therefore, drag force ${F}_{D}$ is considered the main influential force in determining particle trajectories, which is defined,

5 $${F}_{D}=\frac{18\mu }{{\rho }_{p}{d}_{p}^{2}}\frac{{C}_{{\text{D}}}{Re}_{p}}{24},$$

where ${d}_{p}$ and ${\rho }_{p}$ are the particle diameter and density, respectively; ${C}_{{\text{D}}}$ represents the drag coefficient which can be obtained based on Spherical drag law^20^:

6 $${C}_{{\text{D}}}={a}_{1}+\frac{{a}_{2}}{{{\text{Re}}}_{{\text{P}}}}+\frac{{a}_{3}}{{{\text{Re}}}_{{\text{P}}}^{2}}.$$

${a}_{1}$, ${a}_{2}$, and ${a}_{3}$ depend on particle Reynolds number, ${Re}_{p}= \rho {d}_{p}({u}_{p}-\overline{u})/\mu$. Morsi and Alexander^20^ provided the values for different ranges of ${Re}_{p}$. The numerical solver applies the correct values based on the particle Reynolds number.

The continuous and discrete phases are coupled using a one-way approach. This decision was made based on the fact that the volume particle fraction is below ${10}^{-6}$, a threshold below which the particle flow is considered highly diluted. According to the coupling classification map introduced by Ref.^21^, one-way coupling is deemed adequate in this range. In addition, to include the effect of turbulence fluctuations on particle dispersion a stochastic tracking technique with discrete random walk is employed.

**Figure 1:**
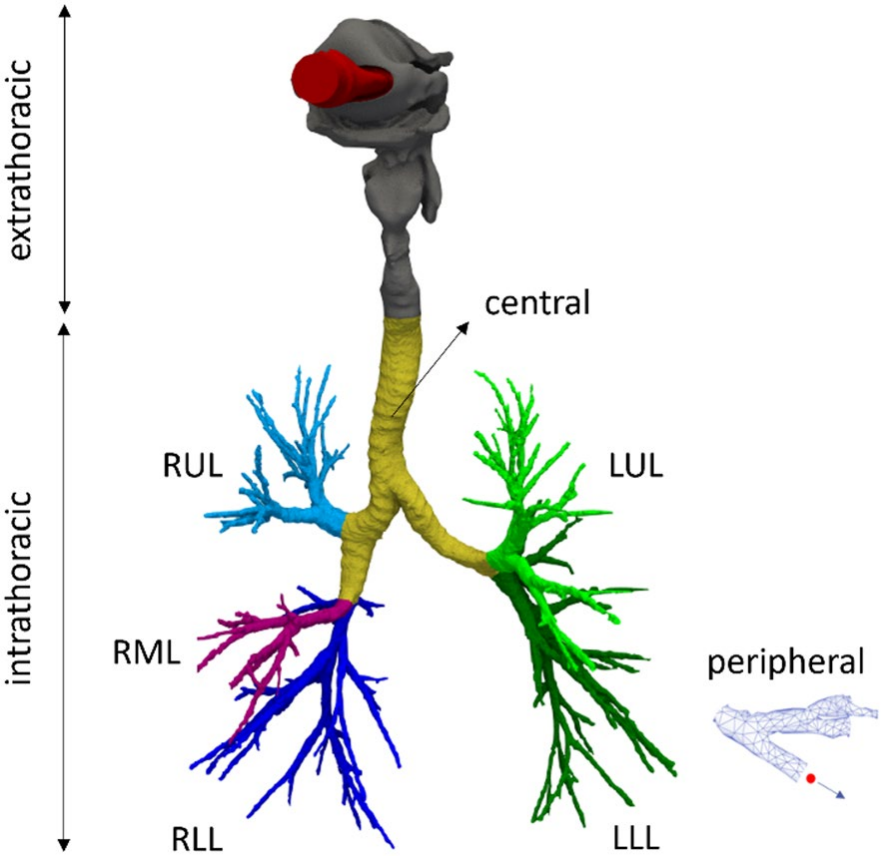
Zones of deposition: extrathoracic (mouth and upper airways, gray), central (trachea and main bronchi, yellow), distal (visible small airways, indigo, purple, blue, light and dark green), peripheral (invisible airway, i.e. doses exiting outlets), intrathoracic (sum of distal and peripheral depositions). Total lobe deposition: aggregate of distal and peripheral depositions on lobe level, right upper lobe (RUL = ${{\text{RUL}}}_{{\text{d}}}$+${{\text{RUL}}}_{{\text{p}}}$), right middle lobe (RML = ${{\text{RML}}}_{{\text{d}}}$+${{\text{RML}}}_{{\text{p}}}$), right lower lobe (RLL = ${{\text{RLL}}}_{{\text{d}}}$+${{\text{RLL}}}_{{\text{p}}}$), left upper lobe (LUL = ${{\text{LUL}}}_{{\text{d}}}$+${{\text{LUL}}}_{{\text{p}}}$), left lower lobe (LLL = ${{\text{LLL}}}_{{\text{d}}}$+${{\text{LLL}}}_{{\text{p}}}$).

The airways of patients are segmented from their CT scans. Figure 1 shows the deposition zones of a representative airways that are grouped into the extrathoracic and intrathoracic regions. The extrathoracic zone consists of the mouth and the upper airways. The intrathoracic region begins at the top of the trachea and extends downward through the rest of the airways. In the intrathoracic airways, yellow area (comprising the trachea and main bronchi) is the central zone. Airways visible on a CT scan, with a diameter above 1 to 2 mm, indicated by indigo, purple, blue, light and dark green colors, constitute the distal zone. The peripheral zone is defined based on airway diameter. The small airways, below 1 to 2 mm in diameter, which cannot be captured on a CT scan are considered as the peripheral zone. The dose of particles exiting the domain from the airway outlets is regarded as the peripheral deposition. An alternative approach is to apply idealized diseased insensitive expansion of airways using algorithms such as space filling growth. Although there is a chance that they add values for ideal cases of healthy subjects, they may fail for most diseased airways due to the variabilities in individual airway geometries for different diseases and their abnormal manifestations^22^. Moreover, as the best of our knowledge there is no method for validating the extrode of those artificial branches up to alveoli.

The lobar deposition—namely, the right upper lobe (RUL), right middle lobe (RML), right lower lobe (RLL), left upper lobe (LUL), and left lower lobe (LLL)—is determined by combining the lobe-specific distal and peripheral deposition portions. An example calculation for RUL is expressed as *RUL* = ${RUL}_{d}$+${RUL}_{p}$, where d and p denote the distal and peripheral deposition components, respectively. The same methodology is applied to the other lobes.

**Figure 2:**
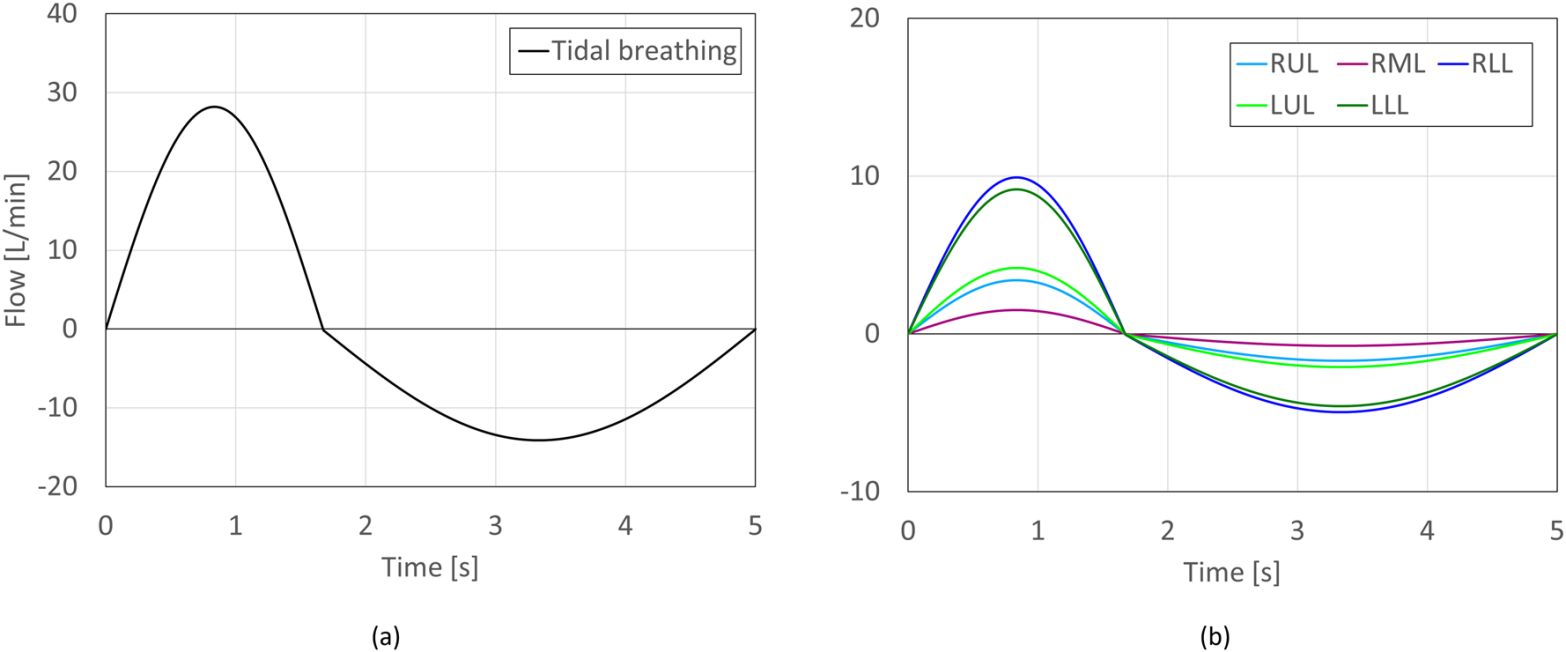
(**a**) Tidal breathing profile and (**b**) patient-lobe-specific flow profiles. For this representative patient, lobar airflow distributions are: RUL: 12.09%, RML: 5.39%, RLL: 35.17%, LUL: 14.86%, and LLL: 32.47%.

The velocity and pressure boundary conditions for the continuous phase are set as follows: At the inlet, Dirichlet boundary conditions are assigned for pressure, while Neumann conditions are applied for velocity. In contrast, at the outlets, Neumann conditions govern the pressure, and patient-specific time-dependent boundary conditions are enforced for velocity. For each patient, in order to mimic a realistic airway ventilation scenario, two CT scans at FRC and TLC are utilized. The differences in the lobe volumes of each capacity level is an indication of the percentage of the air expelled from that lobe. Using this data alongside the established outlet surface areas, the conceptual tidal breathing pattern illustrated in Fig. 2a is transformed into patient-specific lobar flow profiles, as illustrated in Fig. 2b (as a representative of a patient). These profiles are then designated as velocity profiles at the outlets. On the walls, device, and airways, No-slip conditions are imposed for velocity while Neumann conditions are applied for pressure.

Inert particles, with an average diameter of 1.32 µm and a density of 1015 kg/m^3^, were consistently introduced throughout the breathing cycle. An injection dependence analysis was performed (Table 1). Our findings show that there is no significant changes in deposition when the number of particles was larger than 1.2 million. A 'trap' boundary condition governed the device and airway walls, causing deposition upon particle contact with the airway surfaces. This aligns with the anticipated behavior of droplets when they come into contact with surfaces. At the outlets, an 'escape' condition was utilized, enabling particles to exit through the domain outlets upon their arrival. As previously mentioned, this part of the drug dosage is classified as peripheral deposition.

**Table 1:** Influence of the injected particle parcel number on regional deposition as the percentage of delivered dose.

Particles	Intrathoracic	Distal	Peripheral
32,882	69.29	23.15	46.14
81,405	68.85	23.25	45.6
162,810	69.17	23.09	46.07
537,273	68.95	23.22	45.74
814,050	69.05	23.28	45.77
1,084,314	69.06	23.24	45.82
1,221,075	68.96	23.13	45.83
1,628,100	68.97	23.19	45.78
2,035,125	68.99	23.2	45.79
3,256,200	69.01	23.2	45.81

In order to ensure the final numerical results are independent of the grid and timestep chosen, three poly-hexcore meshes, named A, B and C, each at three different time steps $\Delta t \in ((5, 2.5, 1.25)\times {10}^{-3}\mathrm{ s})$, are investigated. The main characteristics of each mesh alongside their correspounding results are summarized in Table 2. In the regions where high speed and/or transitional flow may occur, in extrathoracic and central areas, 3 prism layers (${{\varvec{N}}}_{{\varvec{l}}}$) with constant growth rate of ($\mathbf{r}$) 1.3 are introduced. The height of the first layer on the wall ($\Delta {\varvec{x}}$) reduced as refining the mesh from A to C. The regional lung deposition, expressed as a percentage of the delivered dose (% DD), are regarded as metrics for assessing the sensitivity of the results.

**Table 2:** Regional lung deposition sensitivity to the grid and time step sizes.

Mesh	$N$	${N}_{l}$	$\Delta x$ [mm]	${\text{r}}$	$\Delta \mathrm{t }\times {10}^{-3}$ [s]	Intrathoracic	Central	Distal	Peripheral
A	3,638,985	3	0.04	1.3	$5$	66.78	1.06	19.07	47.72
					$2.5$	66.99	1.08	19.14	47.84
					$1.25$	67.00	1.06	19.07	47.94
B	4,850,981	3	0.015	1.3	$5$	67.01	0.51	18.46	48.55
					$2.5$	67.37	0.50	18.49	48.88
					$1.25$	67.46	0.49	18.50	48.95
C	6,496,934	3	0.01	1.3	$5$	66.70	0.41	18.20	48.51
					$2.5$	66.90	0.39	18.29	48.61
					$1.25$	66.94	0.39	18.22	48.72

$N$ represents the number of cells across the domain; ${N}_{l}$ is the number of prism layer accommodated on the walls, $\Delta x$ denotes the height of the first cell, r accounts for the layer growth rate from the wall and $\Delta t$ indicates the time step. Deposition values on each zone are given as percentages of the delivered dose, dose entering the mouth.

The outcomes across all three meshes and time steps showed remarkably similar results concerning intrathoracic, central, distal, and peripheral depositions. When comparing the deposition levels of mesh A with the time step of $\Delta t=5 \times {10}^{-3}$ against mesh C with the time step of $\Delta t=1.25 \times {10}^{-3}$, the variance was less than one percent. Essentially, mesh A achieved considerable deposition accuracy while operating at approximately half the resolution of mesh C, making it an appropriate choice balancing accuracy and computational demands. Moreover, the impact of reducing the time step appeared negligible. Hence, mesh A with a time step of $\Delta t=5 \times {10}^{-3}$ has been chosen for all subsequent simulations.

## Results and discussion

Six mild or moderate asthma patients with the age range of 23–65 were previously enrolled in a single study described in Ref.^11^. There were three males and three females with an average forced expiratory volume of 102.7% ± 12.8 (standard deviation). The average forced expiratory volume divided by forced vital capacity (Tiffeneau ratio) of the patients was 76.33% ± 7.06. Patients underwent SPECT/CT within 24 h after CT.

**Figure 3:**
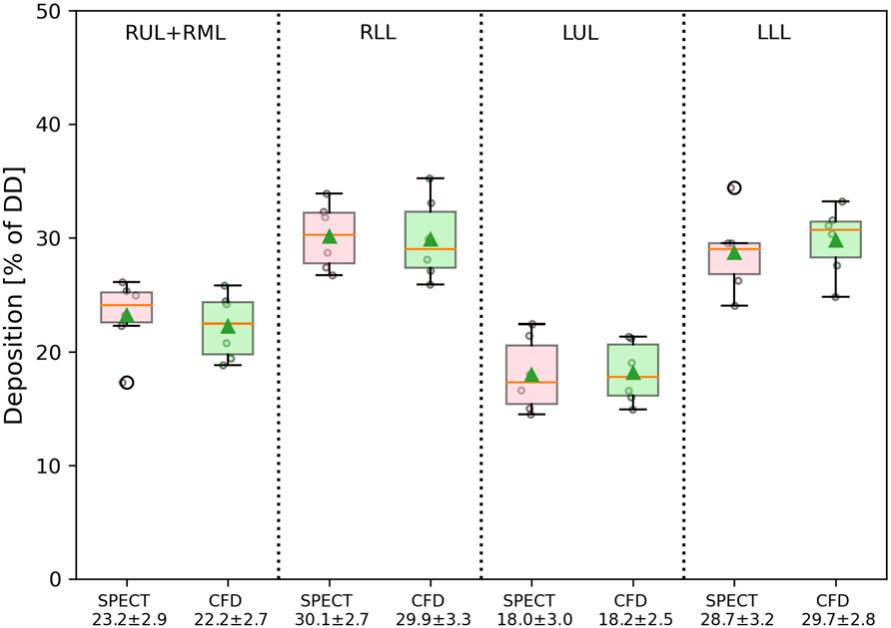
Lobar deposition in six asthma patients obtained from in-vivo SPECT and CFD. % of DD: as a percentage of delivered dose. Lobar depositions: right upper and right middle lobes (RUL + RML), right lower lobe (RLL), left upper lobe (LUL), left lower lobe (LLL).

Figure 3 shows box plots for the in-vivo lobar deposition for the six patients and compares them with the predicted data obtained from the CFD model. In SPECT/CT experiment, it is often challenging to capture the horizontal fissure in the right lung with a good precision due to the low-dose protocol, therefore deposition in RUL and RML were combined. As a result, in CFD calculations same approach is followed. The mean difference, the average of the absolute differences in deposition results for the four zones, between the SPECT data and CFD is 2.10%, rendering a good agreement between these two methods.

Table 3 presents the local deposition as a percentage of the dose deposited in the distal and peripheral zones for each patient. The lobar mean differences of CFD predictions with respect to SPECT shows a good agreement between these two approaches. However some differences are observed. The maximum and minimum differences are for patient 2 (4.12%) and patient 5 (0.82%), respectively. The larger discrepancies in former can be associated with the possible differences in the original tidal breathing pattern of the patient, during SPECT image acquisition, and the idealized flow profile used in CFD, see Fig. 2a. Although this variation can be influential, it is the lobar airflow distribution (obtained from FRC and TLC scans) that mostly determine the fate of particles in the lungs.

**Table 3:** Comparison of deposition data predicted with CFD and the in-vivo SPECT data presented in Ref.^11^.

Patient	Method	RUL/RML	RLL	LUL	LLL	Mean difference with SPECT
1	SPECT	26.13%	27.41%	18.00%	28.47%	
	CFD	24.38%	25.86%	16.59%	33.17%	2.35%
2	SPECT	25.34%	31.84%	16.59%	26.23%	
	CFD	18.78%	35.30%	14.90%	31.02%	4.12%
3	SPECT	17.27%	33.90%	14.47%	34.36%	
	CFD	19.35%	33.07%	16.03%	31.56%	1.82%
4	SPECT	22.33%	26.68%	21.44%	29.55%	
	CFD	24.19%	27.07%	21.16%	27.57%	1.13%
5	SPECT	24.93%	28.68%	22.42%	23.97%	
	CFD	25.73%	28.11%	21.35%	24.81%	0.82%
6	SPECT	23.19%	32.30%	14.98%	29.52%	
	CFD	20.78%	29.93%	19.00%	30.29%	2.39%

Lobar depositions: right upper and right middle lobes (RUL + RML), right lower lobe (RLL), left upper lobe (LUL), left lower lobe (LLL).

**Figure 4:**
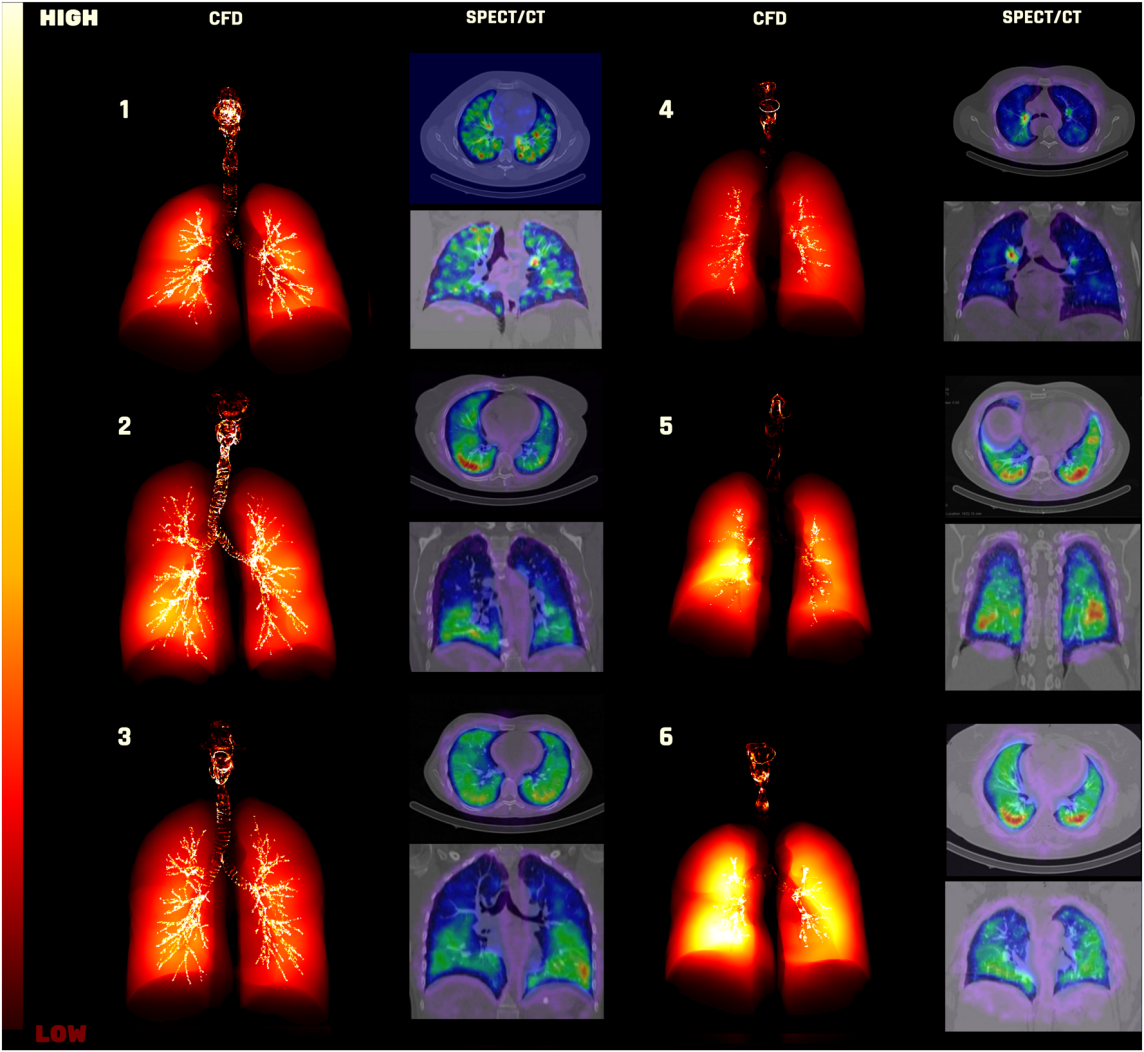
Qualitative comparison of between the predicted deposition and the experimental SPECT data presented in 15. For each patient, the figure on the left is the CFD results and the one on the right is obtained from SPECT.

Figure 4 highlights the locations of high deposition concentration in each patient obtained from CFD, and compares it to the SPECT results. For each patient, the left hand side figures (with a blackbody colormap) are the scintigraphy-like images derived from the CFD models, and the right hand side figure (with a rainbow colormap) is extracted from the SPECT study (top and front views).

An important point about the CFD illustrations for deposition is that the results are not limited to the lung regions within the resolution of the CT scan. Local deposition at the peripheral zones can also be visualized as shown in Fig. 4. The information on the exited aerosols from each branch (i.e. peripheral deposition) and the patient specific geometries of the lobes allow for postprocessing the CFD results and visualize the peripheral deposition as well as the deposition on different airway regions depicted by a CT scan. The hot spots in the SPECT figures of patients 1, 2, 3, and 5 correspond well to the high deposition regions predicted by the CFD models. As such, higher deposition in the lower lobes of patient 2 is highlighted in both approaches. However, it is acknowledged that the comparison is imperfect.

It should be noted that the CFD figures show the cumulative deposition in the perpendicular direction to the planar view, whereas the SPECT figures show a 2D view of the deposition at the plane of focus. Therefore, Fig. 3 and Table 3 are more informative for a more accurate comparison.

In the following, we further highlight the importance of applying patient specific boundary conditions using their regional air distribution as well as the role of anatomical upper airways when predicting lung deposition using CFD. The clinical implications are discussed.

### Influence of applying correct lung ventilation

As discussed in Materials and Method, Functional Respiratory Imaging (FRI) uses patient specific boundary conditions extracted the TLC and FRC scans for individual patients and determined the air volume ventilated from each lobe. To understand the importance of applying patient specific boundary conditions using their FRC and TLC CT scans, Patient 001 of the current study is considered for an additional simulation. As such, quality of expiration of the patient is ignored, and the boundary condition at the outlet of each branch is uniformly set as pressure outlet.

**Figure 5:**
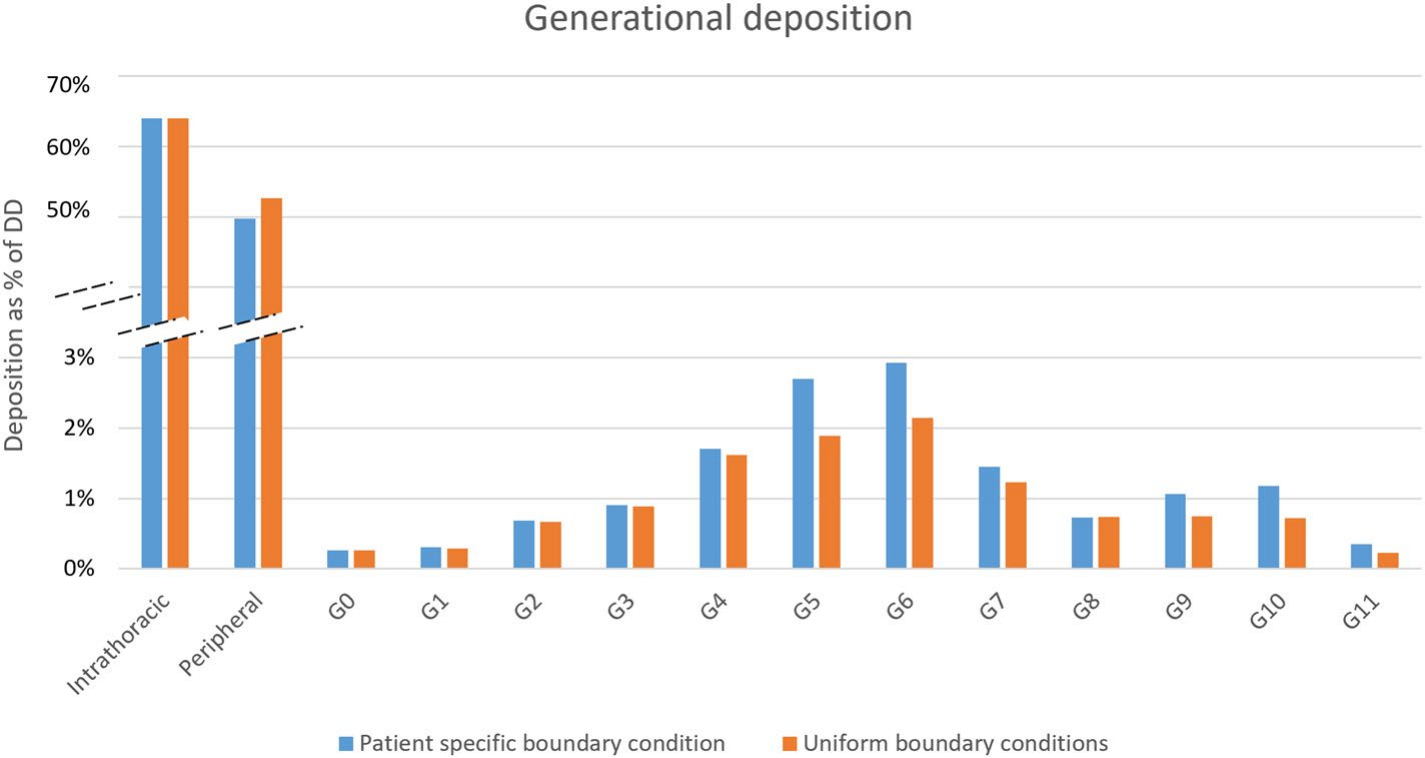
Generational deposition when applying patient specific ventilation conditions and applying a generic boundary condition to all outlets. % of DD: as a percentage of delivered dose.

Figure 5 shows aerosol deposition in the generational level when applying patient specific ventilation conditions versus a uniform pressure outlet boundary condition at airway outlets. Although the intrathoracic deposition are very similar, aerosol deposition differs in all generations from 0 to 11 as well as the peripheral zone. For instance, implementing patient-specific boundary conditions results in a 1.5-fold rise in the localized deposition of generation 5 within the examined airway when contrasted with the uniform pressure outlet boundary condition. This shows in medication dosage reaching the specific area could hold significant importance for drug delivery in pulmonary diseases. Therefore, for aerosol deposition evaluations, it is essential to apply the patient specific boundary condition considering their FRC and TLC scan.

### Influence of patient specific upper airway

**Figure 6:**
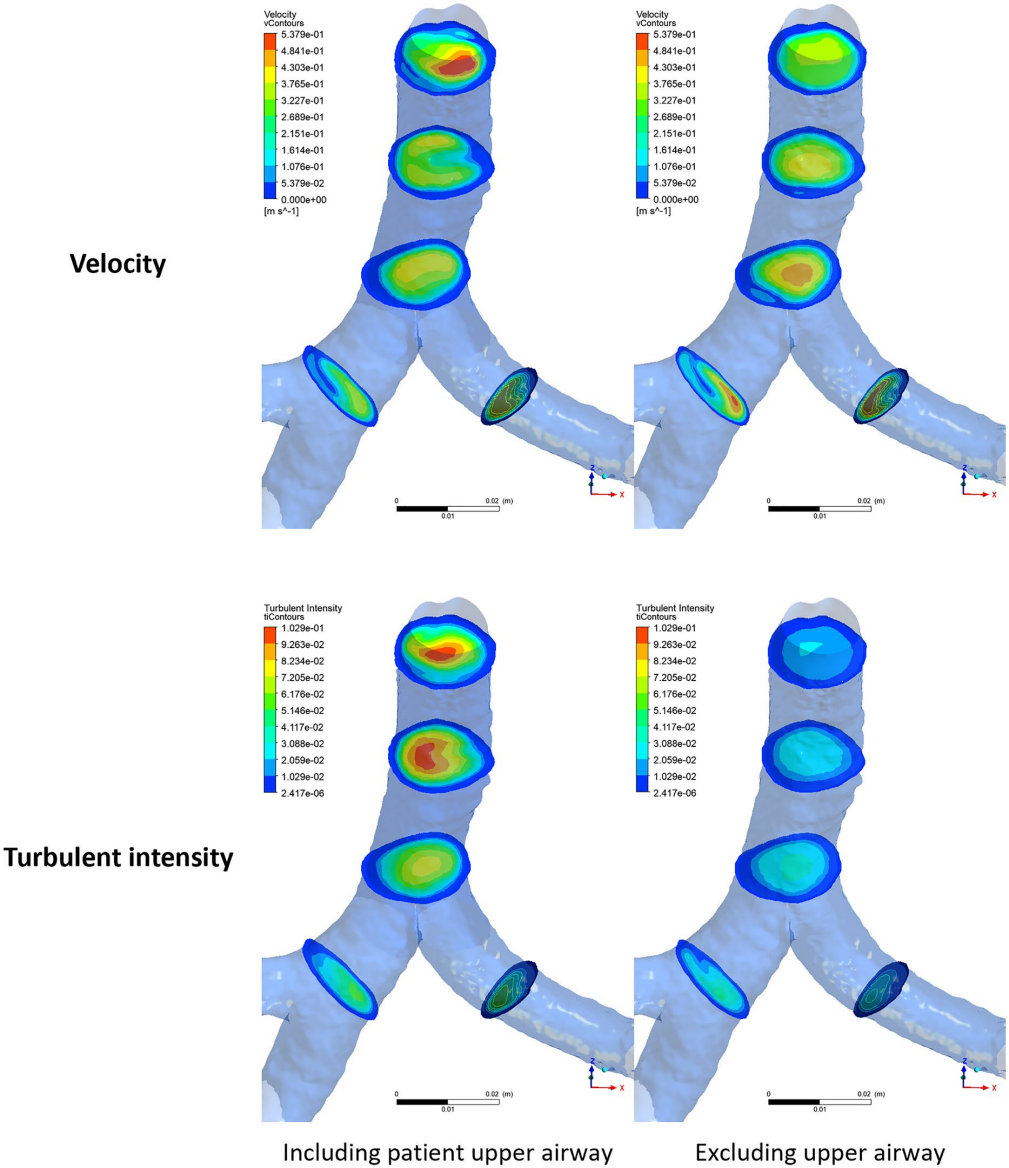
Contours of velocity (top) and turbulent intensity (bottom) for the cases including upper airway (left) and excluding upper airway (right).

Simplifying the model by excluding mouth and upper airway, or removing large particle using idealized algorithms may provide misleading regional deposition. To investigate the influence of mouth and upper airway, an extra simulation is performed excluding those zones. Firstly, the flow profiles are noticeably different with and without upper airways. Figure 6 shows the profiles of velocity and turbulent intensity at the trachea and main bronchi zones. The profiles on the left show the case considering the upper airway and mouth, and the ones on the right show the case when excluding them from simulations. The significant change in the profiles of velocity, pressure and turbulence quantities will naturally influence the aerosol trajectory and consequently medication deposition in lower airways. Focusing on the tracheal region shows that incorporating authentic upper airway and mouth structures captures turbulent flow dynamics in the proximal lower airways, leading to increased aerosol dispersion. This heightened dispersion could potentially impact the deposition of aerosols in these regions.

To further demonstrate the influence of mouth and upper airway geometry, the delivery of identical medication in two airway models was simulated. Two realistic upper airway models, one with a wide larynx and one with a narrow larynx, were segmented from CT scans of historical clinical trial participants. These upper airways were coupled to the same lower airway model derived from the CT scan of a healthy male subject. The same anatomical markers were used for coupling. An inhalation profile with a mean flow of 60 L/min and a dry powder inhaler with median aerodynamic diameter (MMAD), geometric standard deviation (GSD), and fine particle fraction (FPF) of 1.51 µm, 2.23, and 39.6%, respectively were used. All boundary and initial conditions of the simulation were otherwise kept identical.

**Figure 7:**
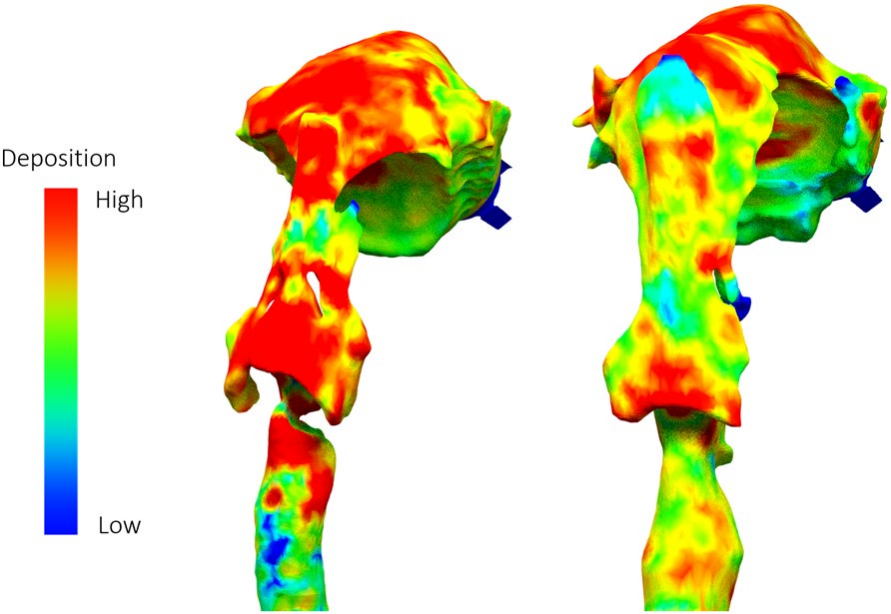
Extrathoracic deposition heatmaps for the two upper airways (UA), left) narrow UA, right) wide UA.

As expected, the simulation yielded intrathoracic deposition of 20.41% (% delivered dose) in the wide-larynx airway model and 10.78% in the narrow-larynx model. The deposition in the central and distal zones of the airway was 5.94% and 3.1% for the wide- and narrow-larynx models, while the peripheral deposition was 14.47% and 7.68%, respectively. Figure 7 shows the heat map of aerosol deposition highlighting the influence of upper airway geometry. Therefore, excluding upper airways or simplifying the model using idealized or simplified aerosol filtration at upper airways may have significant impact on total and regional aerosol deposition.

### Clinical implications

The study clearly demonstrates a high degree of variability and heterogeneity among asthma patients. This variation is evident in the ways the disease affects airway geometry and local air flow, as well as the distribution of inhaled particles in the lungs. If inhalers are prescribed without a detailed understanding of these regional characteristics, essentially treating the lung as a black box, there is a high likelihood of a mismatch between the drug's local deposition and the actual site of the disease in the lungs. Areas that do not receive proper treatment can experience increased air trapping, potentially leading to worsened symptoms. Tools similar to the ones we propose, that provide safe, non-invasive insights into local drug deposition patterns can be instrumental in choosing the most effective medication and inhaler type.

Fortunately, the range of available treatments for asthma is expanding. There are now more drugs offered in various devices than ever before, and biologics are quickly proving to be effective in treating severe asthma. It's only a matter of time before these potent, albeit costly, medications are used for moderate and milder forms of asthma. The availability and expense of these new drugs make a one-size-fits-all approach impractical, necessitating a more personalized evaluation of each patient. The technology discussed in this paper is expected to become a valuable clinical tool for determining the most suitable treatment for a patient at any given time, such as biologics and/or a combination of ICS (inhaled corticosteroid), LABA (long-acting β2-agonist), and LAMA (long-acting muscarinic antagonist). The authors anticipate that this will aid efforts in the asthma field to not only achieve remission but also prevent disease progression by enabling more effective early intervention.

## Conclusion

The predicted deposition of inhaled aerosols in six asthma patients obtained from CFD is compared with the SPECT data of the same population. The predicted results of CFD model compare well with the SPECT data. The importance of anatomical upper airway and patient specific boundary conditions using their inspiratory and expiratory CT scans is highlighted. This study, shows that CFD technique can be a promising tool to simulate patient-specific deposition if correct boundary conditions are applied and can generate similar information obtained with functional imaging tools, such as SPECT. This study seems to be the initial attempt at directly comparing a transient CFD model predictions of aerosol deposition in six asthmatic patients with their own in-vivo data. The study highlights diverse lung characteristics in asthma patients, impacting how drugs are delivered. Such tools are vital for optimizing medication and inhaler selection based on individual lung patterns.

## Data Availability

The datasets used and/or analysed during the current study available from the corresponding author on reasonable request.

## References

[1] Asher, M. I. & Ellwood, P. The global asthma report 2014 (2014).

[2] Conway, J. Lung imaging—Two dimensional gamma scintigraphy, SPECT, CT and PET. *Adv. Drug Deliv. Rev.* **64**, 357–368 (2012). 22310158 10.1016/j.addr.2012.01.013

[3] Leach, C. L., Kuehl, P. J., Chand, R. & McDonald, J. D. Respiratory tract deposition of HFA–beclomethasone and HFA–fluticasone in asthmatic patients. *J. Aerosol Med. Pulm. Drug Deliv.* **29**, 127–133 (2016). 26061801 10.1089/jamp.2014.1199

[4] Dolovich, M. B. Measuring total and regional lung deposition using inhaled radiotracers. *J. Aerosol Med.* **14**, 35–44 (2001). 10.1089/08942680150506321 11424892

[5] Ciciliani, A.-M., Langguth, P. & Wachtel, H. In vitro dose comparison of Respimat® inhaler with dry powder inhalers for COPD maintenance therapy. *Int. J. Chron. Obstruct. Pulmon. Dis.* **12**, 1565 (2017). 28603412 10.2147/COPD.S115886 PMC5457178

[6] Poorbahrami, K., Vignon-Clementel, I. E., Shadden, S. C. & Oakes, J. M. A whole lung in silico model to estimate age dependent particle dosimetry. *Sci. Rep.* **11**, 1–12 (2021). 34045500 10.1038/s41598-021-90509-8 PMC8159973

[7] Wedel, J. *et al.* Anatomy matters: The role of the subject-specific respiratory tract on aerosol deposition—A CFD study. *Comput. Methods Appl. Mech. Eng.* **401**, 115372 (2022). 35919629 10.1016/j.cma.2022.115372 PMC9333481

[8] Williams, J., Kolehmainen, J., Cunningham, S., Ozel, A. & Wolfram, U. Effect of patient inhalation profile and airway structure on drug deposition in image-based models with particle-particle interactions. *Int. J. Pharm.* **612**, 121321 (2022). 34875355 10.1016/j.ijpharm.2021.121321

[9] Atzeni, C. *et al.* Computational fluid dynamic models as tools to predict aerosol distribution in tracheobronchial airways. *Sci. Rep.* **11**, 1109 (2021). 33441807 10.1038/s41598-020-80241-0 PMC7806585

[10] V&V40, A. Assessing credibility of computational modeling through verification and validation: application to medical devices. *Am. Soc. Mech. Eng.* (2018).

[11] De Backer, J. W. *et al.* Validation of computational fluid dynamics in CT-based airway models with SPECT/CT. *Radiology* **257**, 854–862 (2010). 21084417 10.1148/radiol.10100322

[12] Tian, G., Hindle, M., Lee, S. & Longest, P. Validating CFD predictions of pharmaceutical aerosol deposition with in vivo data. *Pharm. Res.* **32**, 3170–3187 (2015). 25944585 10.1007/s11095-015-1695-1 PMC4580521

[13] Kannan, R. R. *et al.* Pharmaceutical aerosols deposition patterns from a dry powder inhaler: Euler Lagrangian prediction and validation. *Med. Eng. Phys.* **42**, 35–47 (2017). 27993478 10.1016/j.medengphy.2016.11.007

[14] Kim, Y. H., Tong, Z. B., Chan, H. K. & Yang, R. Y. CFD modelling of air and particle flows in different airway models. *J. Aerosol Sci.* **134**, 14–28 (2019). 10.1016/j.jaerosci.2019.04.015

[15] Feng, Y. *et al.* An in silico inter-subject variability study of extra-thoracic morphology effects on inhaled particle transport and deposition. *J. Aerosol Sci.* **123**, 185–207 (2018). 10.1016/j.jaerosci.2018.05.010

[16] Feng, Y., Zhao, J., Hayati, H., Sperry, T. & Yi, H. Tutorial: Understanding the transport, deposition, and translocation of particles in human respiratory systems using computational fluid-particle dynamics and physiologically based toxicokinetic models. *J. Aerosol Sci.* **151**, 105672 (2021). 10.1016/j.jaerosci.2020.105672

[17] Menter, F. R. Two-equation eddy-viscosity turbulence models for engineering applications. *AIAA J.* **32**, 1598–1605 (1994). 10.2514/3.12149

[18] Inthavong, K., Choi, L.-T., Tu, J., Ding, S. & Thien, F. Micron particle deposition in a tracheobronchial airway model under different breathing conditions. *Med. Eng. Phys.* **32**, 1198–1212 (2010). 20855226 10.1016/j.medengphy.2010.08.012

[19] Pourmehran, O., Gorji, T. B. & Gorji-Bandpy, M. Magnetic drug targeting through a realistic model of human tracheobronchial airways using computational fluid and particle dynamics. *Biomech. Model. Mechanobiol.* **15**, 1355–1374 (2016). 26886215 10.1007/s10237-016-0768-3

[20] Ansys® FLUENT. Ansys® FLUENT Release 2020.2, Theroy Guide. *ANSYS Inc* (2020).

[21] Elghobashi, S. On predicting particle-laden turbulent flows. *Appl. Sci. Res.* **52**, 309–329 (1994). 10.1007/BF00936835

[22] Pu, J. *et al.* CT based computerized identification and analysis of human airways: A review. *Med. Phys.* **39**, 2603–2616 (2012). 22559631 10.1118/1.4703901 PMC3344883

